# Antimicrobial Resistance in Bacterial Poultry Pathogens: A Review

**DOI:** 10.3389/fvets.2017.00126

**Published:** 2017-08-10

**Authors:** Nguyen Thi Nhung, Niwat Chansiripornchai, Juan J. Carrique-Mas

**Affiliations:** ^1^Oxford University Clinical Research Unit, Hospital for Tropical Diseases, Wellcome Trust Major Overseas Programme, Ho Chi Minh City, Vietnam; ^2^Avian Health Research Unit, Chulalongkorn University, Bangkok, Thailand; ^3^Centre for Tropical Medicine, Nuffield Department of Clinical Medicine, University of Oxford, Oxford, United Kingdom

**Keywords:** antimicrobial resistance, antimicrobials, avian pathogens, poultry production, therapy

## Abstract

Antimicrobial resistance (AMR) is a global health threat, and antimicrobial usage and AMR in animal production is one of its contributing sources. Poultry is one of the most widespread types of meat consumed worldwide. Poultry flocks are often raised under intensive conditions using large amounts of antimicrobials to prevent and to treat disease, as well as for growth promotion. Antimicrobial resistant poultry pathogens may result in treatment failure, leading to economic losses, but also be a source of resistant bacteria/genes (including zoonotic bacteria) that may represent a risk to human health. Here we reviewed data on AMR in 12 poultry pathogens, including avian pathogenic *Escherichia coli* (APEC), *Salmonella* Pullorum/Gallinarum, *Pasteurella multocida, Avibacterium paragallinarum, Gallibacterium anatis, Ornitobacterium rhinotracheale* (ORT), *Bordetella avium, Clostridium perfringens, Mycoplasma* spp., *Erysipelothrix rhusiopathiae*, and *Riemerella anatipestifer*. A number of studies have demonstrated increases in resistance over time for *S*. Pullorum/Gallinarum, *M. gallisepticum*, and *G. anatis*. Among Enterobacteriaceae, APEC isolates displayed considerably higher levels of AMR compared with *S*. Pullorum/Gallinarum, with prevalence of resistance over >80% for ampicillin, amoxicillin, tetracycline across studies. Among the Gram-negative, non-Enterobacteriaceae pathogens, ORT had the highest levels of phenotypic resistance with median levels of AMR against co-trimoxazole, enrofloxacin, gentamicin, amoxicillin, and ceftiofur all exceeding 50%. In contrast, levels of resistance among *P. multocida* isolates were less than 20% for all antimicrobials. The study highlights considerable disparities in methodologies, as well as in criteria for phenotypic antimicrobial susceptibility testing and result interpretation. It is necessary to increase efforts to harmonize testing practices, and to promote free access to data on AMR in order to improve treatment guidelines as well as to monitor the evolution of AMR in poultry bacterial pathogens.

## Introduction

Antimicrobial resistance (AMR) is a worldwide health concern (1). Over recent years a considerable body of evidence highlighting the contribution of antimicrobial usage (AMU) and AMR from animals to the overall burden of AMR has emerged (2). A contributing factor is the excessive use of antimicrobials in food animal production. The magnitude of usage is expected to increase considerably over coming years due to intensification of farming practices in much of the developing world (3). Much of our knowledge and assumptions on the prevalence and evolution of AMR in animal production systems relate to organisms that more often than not are commensal in poultry such as *Escherichia coli* (4–6), *Enterococcus* spp., and *Staphylococcus aureus* (7) as well as foodborne zoonotic pathogens, such as non-typhoidal *Salmonella* (NTS) (5, 8, 9) and *Campylobacter* spp. (10). However, with some exceptions, relatively little is known on the prevalence and mechanisms of AMR in pathogenic bacteria in food animal production, including poultry.

Poultry is one of the most widespread food industries worldwide, and chicken is the most commonly farmed species, with over 90 billion tons of chicken meat produced per year (11). The main reasons are the relatively low production costs and the absence of cultural and religious restrictions for its consumption. A large diversity of antimicrobials are used to raise poultry in most countries (12, 13), mostly through the oral route, with the aim to prevent and to treat disease, but also to enhance growth and productivity (14). A large number of such antimicrobials are considered to be of critical and high importance for human medicine (15).

The indiscriminate use of antimicrobials in animal farming is likely to accelerate the development of AMR in pathogens, as well as in commensal organisms. In addition to the concerns due to the emergence of AMR in bacteria from poultry production, there are also human health concerns about the presence of antimicrobial residues in meat (16) and eggs (17). Additionally, AMR in poultry pathogens is likely to lead to economic losses, derived from the expenditure on ineffective antimicrobials, as well as the burden of untreated poultry disease.

Here, we review and summarize data on phenotypic and genotypic resistance against antimicrobials among known poultry pathogens, in order to identify overall trends and highlight knowledge gaps and methodological issues. This review is intended to act as a baseline to compare country-specific data, as well as an incentive for further isolation and AMR testing of poultry bacterial pathogens using harmonized methodologies.

## Methods

We used the “Web of Knowledge” (www.webofknowledge.com) engine to search for articles published between 2000 and December 2016 containing the terms “AMR” or “antimicrobial susceptibility” in combination with “poultry or chicken,” alongside each one of the following: “*E. coli*,” “*S. pullorum*,” “*S. gallinarum*,” “*Pasteurella multocida*,” “*Avibacterium paragallinarum*,” “*Haemophilus paragallinarum*,” “*Mannheimia haemolitica*,” “*Gallibacterium anatis*,” “*Ornithobacterium rhinotracheale*,” “*Mycoplasma*,” “*Chlamydia psittaci*,” “*Bordetella avium*,” “*Riemerella anatipestifer*,” “*Pseudomonas aeruginosa*,” “*Mycobacterium avium*,” “*Clostridium perfringens*,” and “*Erysipelothrix rhusiophathiae*.” Articles with information on “commensal” *E. coli* and NTS spp. were excluded. Also studies reporting on isolates from healthy animals or meat were excluded. We excluded papers covering bacteria isolated from wildlife and domestic pets.

From each publication containing phenotypic data on AMR, the following information was compiled (where available): (1) type of poultry production (poultry species, broiler chicken, layer chicken, and unspecified type); (2) country location; (3) year of sampling; (4) methodologies employed for AMR testing (including interpretative criteria); and (5) phenotypic resistance data. The prevalence of resistance against specific antimicrobials in individual studies was compiled in tabular form. For papers where dilution methods were used, the MIC50 (or Minimum Inhibitory Concentration required to inhibit the growth of 50% of organisms) were compiled, either as reported or inferred from the MIC distribution. For specific pathogens, AMR prevalence data were summarized by antimicrobial using the median and interquartile range (IQR) across studies. MIC50 data across studies were summarized using the median and IQR for pathogen-antimicrobial combinations investigated in at least three publications. Data summarizing prevalence of AMR was plotted for comparative purposes using spider charts using the *fmsb* package in R software (www.r-project.org).

### *Escherichia* *coli*

*Escherichia coli* is a Gram-negative, facultative anaerobe bacterium of the Enterobacteriaceae family. Since *E. coli* is ubiquitous in the gastrointestinal tract of warm-blooded animals, it has been extensively used to monitor AMR in food animals (including poultry) (18, 19). In addition, some *E. coli* strains hosted by poultry are potential source of AMR genes that may transmit to humans (20, 21).

Certain *E. coli* strains, designated as “avian pathogenic *E. coli*” (APEC) are causative agents of colibacillosis, one of the principal causes of morbidity and mortality in poultry worldwide (22). Only studies investigating APEC strains are included in this review, therefore excluding studies on *E. coli* from chicken enteric samples (23, 24).

A total of 12 publications investigated phenotypic resistance in a total of 1,331 APEC isolates from diseased chickens, from Asia (25–29), Africa (30–32), the United States (33, 34), Spain (35), and Brazil (36) (Table 1). All studies included APEC isolates from the chicken species, except one study that, in addition, included isolates from ducks and geese (26). All studies were carried out using the disk diffusion test, except two where isolates were tested using microbroth dilution, and one that used the broth dilution test (Table 1). Two studies reported the MIC distribution of investigated strains (33). A study on 100 APEC strains from Iran reported 99% resistant strains against colistin using the disk diffusion test (28), but it is not clear what breakpoints were used. In addition, colistin resistance cannot be reliably estimated using disk sensitivity tests (37).

**Table 1:** Summary of results of 12 phenotypic studies on antimicrobial resistance of avian pathogenic *E. coli*.

Study	Reference (country)	Year of study	No. isolates (animal host)	Testing method	Interpretation criteria	Phenotypic resistance
1	(25) (China)	2004–2005	70 (chicken)	Broth dilution	CLSI M31-A2 (2002)	AMP (83.0%), CEF (7.0%), CN (44.0%), S (42.0%), AK (12.0%), C (79.0% R), FFN (29.0%), OTC (100%), SXT (100%), ENR (83.0%), CIP (81.0%)
2	(26) (China)	2007–2014	243 (chickens, ducks geese)	Disk diffusion	CLSI M100-S22 (2012)	AMP (81.1% R; 13.9% I), CTX (21.0% R; 1.0% I), CRO (18.0% R; 2.0% I), CAZ (10.0% R; 5.0% I), ETP (0%), ATM (15.0% R; 2.0% I), CN (28.0% R; 69.0% I), KA (31.0% R; 67.0% I), S (79.0% R; 5.0% I), AK (6.0% R; 2.0% I), TE (97.5% R), SXT (78.2% R), SMX (78.2% R), SSZ (80.7% R), CIP (63% R; 6% I), NA (82.3% R), C (48.0% R; 7.0% I), NIT (13.0% R; 24.0% I)
3	(33) (United States)	2001–2003	445 (chickens)	Broth micro dilution^a^	CLSI M31-A2 (2002) and NARMS (2003)	AMP (40.0% R; 1.6% I), AMC (12.4% R; 11.9% I), CFN (22.2% R; 31.2% I), CEF (3.8% R; 1.8% I), TIC (30.1% R; 6.7% I), CN (44.0% R; 4.3%), SPC (51.5% R; 1.1% I), TIM (98.2% R; 1.1% I), FFN (12.8% R; 62.7% I), TE (79.3% R; 0.2% I), SXT (9.0% R), ENR (3.4% R; 8.3% I), DIF (8.3% R; 9.4% I), ORB (2.5% R; 5.6% I)
4	(27) (Jordan)	Not specified	18 (broilers)	Broth micro dilution	NCCLS M7-A3 (1999)	AMX (100%), CA (100%), CN (41.0%), SPC (47.0%), ERY (100%), FFN (53.0%), OTC (100%), DOX (100%), CIP (71.0%), ENR (76.0%), FOM (35.0%)
5	(30) (Zimbabwe)	2011–2012	103 (chickens)	Disk diffusion^a^	CLSI M100-S17 (2007)	AMP (94.1% R; 1% I), CLX (100% R), CN (1.0% R; 1.9% I), NEO (54.4% R; 37.9% I), TE (100% R), CIP (0% R; 0% I), C (36.9% R; 45.6% I), BAC (100% R)
6	(31) (Egypt)	2014–2016	116 (broilers)	Disk diffusiona	CLSI M100-S20 (2010)	AMP (100%), CTX (58.6%), CN (48.3%), KA (69.0%), S (50.0%), C (84.5%), TE (93.1%), SXT (58.6%), NA (84.5%), CIP (41.4%)
7	(32) (Egypt)	2011	73 (broilers)	Disk diffusion^a^	CLSI M31-A3 (2008)	AMP (97.3%), AMC (35.6%), CFN (65.8%), FOX (61.6%), OXA (78.1%), CTT (60.3%), CTX (23.3%), CPD (21.9%), CO (19.2%), ATM (41.1%), S (93.2%), KA (89.0%), SPC (95.9%), CN (43.8%), C (79.5%), TE (95.9%), SXT (82.2%), NA (67.1%), CIP (15.1%)
8	(36) (Brazil)	2012–2014	15 (broilers)	Disk diffusion^a^	CASFM (document not specified)	AMX (74.0%), AMC (13.0%), CFN (53.0%), CEF (40.0%), FOX (7.0%), CN (20.0%), NEO (7.0%), APR (0%), TE (40.0%), TMP (34.0%), SXT (34.0%), NA (77.0%), FLM (80.0%), ENR (40.0%), COL (0%)
9	(28)^b^ (Iran)	Not specified	100 (chickens)	Disk diffusion^a^	CLSI (document not specified)	CFX (50.0% R; 34.0% I), CN (17.0% R; 2.0% I), OTC (96.0% R; 1.0% I), DOX (95.0% R; 2.0% I), SXT (89.0% R; 1.0% I), NA (100% R), CIP (91.0% R; 2.0% I), NOR (88.0% R; 3.0% I), COL (99.0% R; 1.0% S); LIP (53.0% R; 6.0% I)
10	(29) (Thailand)	2007–2010	50 (chickens)	Disk diffusion^a^	NCCLS M31-A2 (2002)	AMP (82.0% R; 18.0% I), AMX (86.0% R; 14.0% I), AMC (28.0% R; 14.0% I), CLX (72.0% R; 28.0% I), CN (24.0% R; 12.0% I), KA (28.0% R; 10.0% I), NEO (62.0% R; 28.0% I), ERY (80.0%; 20.0%), LCM (94.0% R; 6.0% I), LIP (30.0% R; 42.0% I), TIA (100% R), TIM (100% R), TYL (100% R), TE (32.0% R; 26.0% I), OTC (50.0% R; 8.0% I), DOX (30.0% R; 18.0% I), SXT (34.0% R; 6.0% I), ENR (24.0% R; 6.0% I), NOR (20.0% R; 10.0% I), COL (24.0% R; 10.0% I), FOM (8.0% R; 8.0% I)
11	(35) (Spain)	2012	22 (chickens)	Disk diffusion^a^ broth microdilution	EUCAST (2015)	AMP (78.0%), CTX (34.0%), CAZ (31.0%), FOX (13.0%), CFP (0%), S (69.0%), KA (19.0%), CN (16.0%), C (13.0%), FFN (6.0%), TE (91.0%), SXT (63.0%), TMP (59.0%), NA (88.0%), CIP (91.0%), COL (0%)
12	(34) (United States)	1998–2002	80 (chickens)	Disk diffusion	NCCLS M100-S6 (1995), M2-A6 (1997), M31-A2 (2002)	AMP (11.2%), CEF (8.5%), CN (33.8%), AK (0%), SPC (88.8%), TE (67.5%), SDX (92.5%), SXT (5.0%), ENR (5.1%)

*In studies where intermediate susceptibility is given, results are presented as: R, resistant; I, intermediate resistant*.

*CASFM, Comite De L’antibiogramme De La Societe Francaise De Microbiologie; CLSI, Clinical Laboratory Standard Institute; NCCLS, National Committee for Clinical Laboratory Standards; EUCAST, European Committee on Antimicrobial Susceptibility Testing; AK, amikacin; AMX, amoxicillin; AMC, amoxicillin/clavulanic acid; AMP, ampicillin; APR, apramycin; ATM, aztreonam; BAC, bacitracin; C, chloramphenicol; CA, clavulanic acid; CAZ, ceftazidime; CEF, ceftiofur; CFN, cephalothin; CFP, cefepime; CFX, cefuroxime; CIP, ciprofloxacin; CLX, cephalexin; CN, gentamicin; COL, colistin; CPD, cefpodoxime; CRO, ceftriaxone; CTX, cefotaxime; CTT, cefotetan; DIF, difloxacin; DOX, doxycycline; ENR, enrofloxacin; ERY, erythromycin; ETP, ertapenem; FFN, florfenicol; FLM, flumequine; FOM, fosfomycin; FOX, cefoxitin; KA, kanamycin; LCM, lincomycin; LIP, lincospectin; NA, nalidixic acid; NEO, neomycin; NIT, nitrofurantoine; NOR, norfloxacin; ORB, orbifloxacin; OTC, oxytetracycline; OXA, oxacillin; S, streptomycin; SDT, sulfadiazine/trimethoprim; SDX, sulfadimethoxine; SDZ, sulfadiazine; SMX, sulfamethoxazole; SPC, spectinomycin; SSZ, sulfisoxazole; SXT, co-trimoxazole; TE, tetracycline; TIA, tiamulin; TIC, ticarcillin; TIM, tilmicosin; TMP, trimethoprim; TYL, tylosin*.

*^a^MIC distributions reported; disk concentrations reported*.

*^b^Excluded from summary estimates of resistance*.

Results of phenotypic resistance in APEC are presented in Table S1 in Supplementary Material. Resistance levels of strains were: ampicillin (median 82.0%; IQR 59.0–95.7%), amoxicillin (80.0%; IQR 43.0–93.0%), ceftiofur (8.5%; IQR 5.4–52.9%), streptomycin (69.0%; IQR 46.0–86.0%), gentamicin (30.9%; IQR 18.5–43.9%), kanamycin (31.0%, IQR 23.5–79.0%), chloramphenicol (63.5%, IQR 36.9–79.5%), florfenicol (20.9%; IQR 9.4–41.0%), tetracycline (91.0%; IQR 53.7–96.7%), co-trimoxazole (60.8%; IQR 34.0–85.6%), nalidixic acid (83.0%, IQR 77.0–88.0%), ciprofloxacin (67.0%; IQR 28.3–86.0%), and enrofloxacin (32.0% IQR 5.1–76.0%).

A study from China identified *floR, cmlA, cat1, cat2*, and *cat3* (genes associated with florfenicol and chloramphenicol resistance) among APEC strains (25). In another study from the same country, the presence of class I integrons on isolates from the same country was strongly correlated with multi-drug resistance (93.3% MDR strains were positive for class 1 integron, compared with 12.5% among non-MDR strains) (26).

In a study from Egypt integrons (mostly class 1) were detected in 29.3% isolates, and were associated with the presence of genes encoding for resistance to trimethoprim (*dfrA1, dfrA5, dfrA7, dfrA12*), streptomycin/spectinomycin (*aadA1, aadA2, aadA5, aadA23*), and streptothricin (*sat2*). Other, non-integron-associated resistance genes, included tetracycline (*tet*A and *tet*B), ampicillin (*bla*_TEM_), chloramphenicol (*cat1*), kanamycin (*aphA1*), and sulfonamide (*sul1* and *sul2*). The S83L mutation in the *gyrA* gene (present in 23.2% isolates) was the most frequently genetic determinant of quinolone resistance, followed by *qnrA, qnrB*, and *qnrS* genes (31). A previous study on 73 APEC strains from the same country (of which 67.0% were nalidixic resistance, 15.1% ciprofloxacin resistance), plasmid-mediated quinolone resistance genes *qnrA1, qnrB2, qnrS1* were found in 64.0% isolates, and the fluoroquinolone-modifying acetyltransferase gene (*aac*(*6_*)*-Ib-cr*) in 7.0% isolates (32). However, the study did not investigate quinolone resistance encoded by genetic mutations.

A study on 116 APEC isolates from broilers in Egypt showed a remarkably high percentage of ESBL-producing strains (58.6%). The *bla*_TEM_ and *bla*_CTX−M−1_ genes were the most prevalent genes in these strains (31). In a study from Spain of 11 cephalosporin resistant isolates, 6 contained *bla*_CTX-M-14_, 2 *bla*_SHV-12_, 2 *bla*_CMY-2_, and 1 *bla*_SHV-2_ (35).

A recent study on a large collection (980) of APEC isolates from several countries identified the plasmid-mediated *mcr-1* colistin resistance gene in 8 isolates from China (of 31 tested) and 4 from Egypt (of 20 tested). Most such strains were multi-resistance to 10 or more antimicrobials (38).

A study on APEC isolates from Jordan investigated the most effective synergistic effects of combinations of 11 antimicrobials by calculation of a fractional inhibitory concentration index of checkerboard titrations. The combinations of amoxicillin–clavulanic acid, ciprofloxacin–fosfomycin, oxytetracycline–erythromycin, oxytetracycline–florfenicol, amoxicillin–gentamicin, oxytetracycline–spectinomycin, and spectinomycin–erythromycin were the most effective *in vitro* (27).

### *S*. Pullorum/Gallinarum

*Salmonella* Pullorum/Gallinarum are biovars within the genus *S. enterica* subspecies *enterica* within the family Enterobacteriaceae. They are the etiological agents of pullorum disease (*S*. Pullorum) and fowl typhoid (*S*. Gallinarum), two septicemic diseases widely common in much of the world, though they have been eradicated from commercial poultry operations in many developed countries (39, 40).

Eight publications investigated phenotypic resistance in a total of 780 *S*. Pullorum/Gallinarum isolates from Korea (four publications) (41–44), India (two) (45, 46), Brazil (one) (47), and China (one) (48) (Table 2). All studies used the disk diffusion test, except one study where agar dilution was used (44), and one where both tests were used (41).

**Table 2:** Summary of results of 7 phenotypic studies on antimicrobial resistance of *S*. Pullorum/Gallinarum from poultry.

Study	Reference (country)	Year of study	No. isolates (bacterial spp.)	Testing method	Interpretation	Phenotypic resistance
1	(41) (Korea)	1995–2001	258 (SG)	Disk diffusion test^a^; agar diffusion^b^	NCCLS M31-A (2000)	AMP (13.0%), AMC (3.9%), CN (43.4%), KA (69.6%), TE (74.8%), OTC (77.9%), SXT (1.5%), COL (0.4%), ENR (6.5%), CIP (10.9%), NOR (52.5%), OFL (82.6%)
2	(43) (Korea)	2010–2012	26 (SG)	Disk diffusion test^a^	CLSI M100-s22 (2012)	AMP (0%), CFZ (0%), AMC (0%), CFN (0%), FOX (0%), CTX (0%), IMP (0%), CN (0%), S (0% R; 88.5% I), AK (0%), ERY (100% R), TE (0%), SXT (0%), C (0%), CIP (0%), ENR (0%), NOR (0%)
3	(44) (Korea)	2002–2007	105 (SG)	Agar dilution test	CLSI M31-A2 (2002), CLSI^c^ (2006), DAMR (2006)	AMP (41.9% R), AMX (24.8% R), S (54.3% R), CN (45.7% R), NEO (7.8% R), TE (16.2% R), SMX (36.2% R), NA (98.1% R), ENR (10.5% R; 83.8% I)
4	(47) (Brazil)	2006–2013	32 (SP/SG)	Disk diffusion^a^	CLSI M31-A2 (2002); CLSI M100-S23 (2013)	AMC (0%), CTX (0%), IMP (0%), CAZ (0%), CFP (0%), ETP (0%), CEF (0%), TE (6.3% R), C (0%), FFN (0%), SXT (1%), NA (37.5%), CIP (34.4%), ENR (25.0%)
	(47) (Brazil)	2006–2013	18 (SP)	Disk diffusion^a^	As above	AMC (0%), CTX (0%), IMP (0%), CAZ (0%), CFP (0%), ETP (0%), CEF (0%), ETP (0%), TE (0%), C (0%), FFN (0%), SXT (0%), NA (38.9%), CIP (33.3%), ENR (5.6%)
5	(48) (China)	1962–2010	337 (SP)	Disk diffusion^a^	CLSI M100-S22 (2012)	AMP (34.4%), CAB (25.5%), CFM (46.6%), CTX (2.4%), S (61.7%), CN (5.3%), KA (3.9%), SPC (45.0%), C (4.1%), TE (58.7%), SMX (52.8%), TMP (82.8%), SXT (49.4%), NA (69.0%), CIP (4.5%), NIT (26.4%)
6	(45) (India)	Not given	4 (SG)	Disk diffusion^a^	CLSI M100-S25 (2013)	AMP–SBT (0%), AMC (0%), CRO (0%), AK (0%), S (0%), CN (0%), TE (0%), DOX (0%), ERY (0%), AZI (0%), C (0%), SXT (0%), CIP (0%), ENR (0%), COL (0%)
7	(46) (India)	2009–2010	12 (SG)	Disk diffusion^a^	Disk manufacturer	AMP (8.3% R; 33.4% I), AMC (0% R; 8.3% I), CLX (0% R; 41.7% I), CN (0% R; 8.3% I), KA (0% R; 66.7% I), NEO (0% R; 58.3% I), TE (16.7% R; 83.3% I), ERY (100% R), C (0% R; 33.4% I), SXT (0% R; 33.4% I), NA (75.2% R; 33.4% I), ENR (0% R; 25.0% I), CIP (0% R; 16.7% I), OFL (0% R; 25.0% I), COL (0% R; 16.7% I)

*In studies where intermediate susceptibility is given, results are presented as: R, fully resistant; I, intermediate resistant; SP, S. Pullorum; SG, S. Gallinarum*.

*CLSI, Clinical Laboratory Standard Institute; DAMR, Danish Integrated Antimicrobial Resistance Monitoring and Research Program Use of Antimicrobial Agents and Occurrence of Antimicrobial Resistance in Bacteria from Food Animals, Foods and Humans in Denmark. ISSN 1600-2032; NCCLS, National Committee for Clinical Laboratory Standards; AMP, ampicillin; AMP-SBT, ampicillin–sulbactam; AMX, amoxicillin; AK, amikacin; AMC, amoxicillin/clavulanic acid; AZI, azithromycin; C, chloramphenicol; CAB, carbenicillin; CAZ, ceftazidime; CEF, ceftiofur; CFM, cefamandole; CFN, cephalothin; CFP, cefepime; CFZ, cefazolin; CN, gentamicin; CIP, ciprofloxacin; CLX, cephalexin; COL, colistin; CRO, ceftriaxone; CTX, cefotaxime; DOX, doxycycline; ENR, enrofloxacin; ERY, erythromycin; ETP, ertapenem; FFN, florfenicol; FOX, cefoxitin; IMP, imipenem; KA, kanamycin; NA, nalidixic acid; NEO, neomycin; NIT, nitrofurantoine; NOR, norfloxacin; OFL, ofloxacin; OTC, oxytetracycline; S, streptomycin; SMX, sulfamethoxazole; SMZ, sulfamethoxazole; SPC, spectinomycin; SXT, co-trimoxazole; TE, tetracycline; TMP, trimethoprim*.

*^a^Disk concentrations reported*.

*^b^MIC50 reported*.

*^c^Performance standards for antimicrobial susceptibility testing; sixteenth informational supplement*.

Overall levels of phenotypic resistance were: ampicillin (median 13.0%; IQR 4.1–38.1%), amoxicillin plus clavulanic acid (0%; IQR 0–0%), cefotaxime (0%; IQR 0–1.2%), streptomycin (27.0%; IQR 0–58.0%), gentamicin (2.6%; 0–43.4%), chloramphenicol (0%; IQR 0–0%), tetracycline (11.2%; IQR 0–37.7%), co-trimoxazole (0%; IQR 0–1%), nalidixic acid (69.0%; IQR 38.2–86.6%), ciprofloxacin (2.0%; 0–33.0%), enrofloxacin (2.8%; IQR 0–10.5%).

A study from Korea reported an increase over time in phenotypic resistance among *S*. Gallinarum isolates: whereas in 1995 all isolates were fully susceptible to 12 antimicrobials, except for tetracyclines (>83% resistance), by 2001, levels of resistance were: ampicillin (87.0%), gentamicin (56.6%), kanamycin (30.4%), enrofloxacin (93.5%), ciprofloxacin (89.1%), norfloxacin (47.5%), and ofloxacin (17.4%) (41). Over the same period, the MIC range for enrofloxacin, ciprofloxacin, norfloxacin, ofloxacin also increased considerably, in parallel with an increase in the rate of mutations of the *gyrA* (from 5.6 to 89.1%) (42).

A further study from the same country unexpectedly identified *S*. Gallinarum in table eggs from healthy chicken layer flocks (43). Surprisingly, isolates were pan-susceptible for most antimicrobials except for streptomycin (88.5% were intermediate resistance).

A study of 42 quinolone resistant strains identified the substitution of a Ser to a Phe or Tyr at position 83 in the *gyrA* gene among 71.0% of isolates. The study identified three different class 1 integrons among 57 sulfonamide resistant strains, containing resistance genes *aadA* (52.6%), *aadB* (12.3%), or *aadB-aadA*. In addition, isolates harboring the integron containing *aadB*-*aadA* displayed resistance to aminoglycosides, as well as increased resistance to fluoroquinolones. As in the case of *E. coli* strains, it is suspected that integrons are largely responsible for multi-drug resistance; clonal expansion and horizontal gene transfer may have contributed to the spread of AMR integrons in these organisms (49).

A study of 337 *S*. Pullorum strains from China showed a consistent increase in resistance between 1962 and 2010. Resistance levels against 11 of 16 antimicrobials tested was significantly greater among int1(+) than int1(−) isolates, and resistance levels to cefamandole, trimethoprim and co-trimoxazole were significantly higher for biofilm-positive types compared with the biofilm-negative groups (48). Recently, full genome sequencing of a multi-drug resistant *S*. Pullorum isolate from China has been published, and included two prophages, the ST104 and prophage-4 (Fels2) previously found in *E. coli* (50).

### *Pasteurella* *multocida*

*Pasteurella multocida* is a Gram-negative, non-motile, facultative anaerobic bacterium of the Pasteurellaceae family. It is the causative agent of fowl cholera, a disease that often manifests as acute fatal septicemia in adult birds, although chronic, and asymptomatic infections also occur (51).

A total of eight publications have investigated phenotypic resistance in *P. multocida* isolates, including studies from the United States (33, 52), Brazil (53, 54), India (55), Indonesia (56), Hungary (57), and Egypt (58) (Table 3). Four studies investigated isolates originating exclusively from the chicken species, and the other four included isolates from ducks, geese, Muscovy ducks, pheasants, and quails, in addition to chicken isolates. Five studies used disk diffusion, and three the broth microdilution technique.

**Table 3:** Summary of results of 8 phenotypic studies on antimicrobial resistance of *P. multocida* from poultry.

Study	Reference (country)	Year of study	No. isolates (host species)	Testing method	Interpretation	Phenotypic resistance
1	(55) (India)	Not given	94 (chicken), 22 (duck), 4 (quail), 2 (turkey), 1 (goose)	Disk diffusion^a^	Disk manufacturer	PEN (49.6% R; 43.9% I), AMP (23.6% R; 22.8% I), CAB (59.3% R; 26.0% I), CN (23.6% R; 20.3% I), S (32.5% R; 44.7% I), AK (55.5% R; 19.5% I), C (6.5% R; 19.5% I), ERY (50.4% R; 49.6% I), LCM (2.4% R; 35.8% I), TE (24.4% R; 43.1% I), OTC (8.1% R; 30.1% I), DOX (25.2% R; 17.9% I), SDZ (100% R), TMP (39.0% R; 9.8% I), SXT (31.7% R; 13.8% I), CIP (8.9% R, 40.6% I), ENR (8.1% R; 20.3% I), NOR (8.1% R; 30.1% I), RIF (44.5% R; 25.2%), NIT (34.1% R; 26.0% I)
2	(56) (Indonesia)	1998–1999	9 (chicken)	Disk diffusion^a^	Not indicated	AMP (0% R), CN (11.1% R, 11.1% I), S (22% R, 66.7% I), ERY (22% R, 77.8% I), LCM (100% R), TE (55.6%R; 11.1% I), DOX (11.1% R, 11.1% I), SDZ (100% R), TMP (0% R), ENR (22.2% R, 11.1% I), BAC (44.4% R, 33% I)
3	(53) (Brazil)	Not given	56 (chicken, turkey)	Disk diffusion^a^	CLSI M31-A3 (2008)	AMX (1.8%), CEF (1.8%), CN (1.8%), ERY (5.4%), ENR (23.8%), TE (12.5%), SQN (76.8%), SXT (19.6%)
4	(58) (Egypt)	Not given	10 (chicken)	Broth microdilution^a^	NCCLS M31-A2 (2006)	AMX (100%), S (0%), FFN (0%), TE (100%), DOX (40%), SXT (0%), CIP (0%)
5	(33) (United States)	2001–2003	80 (chicken)	Broth microdilution^a^	CLSI M31-A2 (2002), NARMS (2006)	AMC (1.2% R), AMP (1.2% R), TIC (0% R), CFN (1.2% R), CEF (1.2% R), CN (2.5% R), SPC (1.2% R), TIM (2.5% R), FFN (1.2% R), TE (6.2% R), SXT (0% R), ENR (1.2% R), DIF (1.2% R), ORB (1.2% R)
6	(57) (Hungary)	2005–2008	7 (geese), 7 (duck), 1 (muscovy duck), 3 (turkey), 1 (chicken), 1 (pheasant)	Disk diffusion^a^	NCCLS M2-A8 (2003)	PEN (0% R), CQN (0% R), APR (15.0% R; 40.0% I), NEO (15.0% R), ERY (0% R; 40.0% I), TUL (0% R), C (0% R), FFN (0% R), TE (15.0% R; 5% I), DOX (0% R; 5% I), FLM (40.0% R), ENR (0% R), OXO (40.0% R), SXT (20.0% R), COL (0% R)
7	(54) (Brazil)	Not given	99 (chicken), 13 (Japanese quail)	Disk diffusion^a^	CLSI M31-A3 (2008)	AMP (3.4% R), CFN (1.6% R), AK (1.6% R), TE (5.1% R)
8	(52) (United States)	2006–2011	207 (chicken)	Broth microdilution	CLSI M31-A2 (2002)	PEN (16.0% R; 16.0% I), AMX (5.0% R; 2.0% I), CEF (3.0% R; 2.0% I), NEO (2.0% R; 9.0% I), CN (6.0% R; 15.0% I), ERY (18.0% R; 78.0% I), TYL (97.0% R; 2.0% I), CLD (97.0% R; 3.0% I), FFN (2.0% R; 4.0% I), TE (9.0% R; 5.0% I), DOX (1.0% R; 0% I), OTC (9.0% R; 5.0% I), STZ (5.0% R; 3.0% I), SDX (9.0% R; 6.0% I), SXT (2.0% R; 2.0% I), ENR (1.0% R; 6.0% I)

*In studies where intermediate susceptibility is given, results are presented as: R, fully resistant; I, intermediate resistant*.

*CLSI, Clinical Laboratory Standard Institute; NCCLS, National Committee for Clinical Laboratory Standards; AK, amikacin; AMC, amoxicillin/clavulanic acid; AMP, ampicillin; AMX, amoxicillin; APR, apramcyn; BAC, bacitracin; C, chloramphenicol; CAB, carbenicillin; CEF, ceftiofur; CFN, cephalotin; CIP, ciprofloxacin; CLD, clindamycin; CN, gentamicin; COL, colistin; CQN, cefquinome; DIF, difloxacin; DOX, doxycycline; ENR, enrofloxacin; ERY, erythromycin; FFN, florphenicol; FLM, flumequine; LCM, lincomycin; NEO, neomycin; NOR, norfloxacin; NIT, nitrofurantoine; ORB, orbifloxacin; OTC, oxytetracycline; OXO, oxolinic acid; PEN, penicillin; RIF, rifampicin; S, streptomycin; SDZ, sulfadiazine; SDX, sulfadimethoxine; SPC, spectinomycin; SQN, sulfaquinoxaline; STZ, sulfathiazole; SXT, co-trimoxazole; TE, tetracycline; TIC, ticarcillin; TIM, tilmicosin; TMP, trimethoprim; TYL, tylosin; TUL, tulathromycin*.

*^a^MIC distributions reported; disk concentrations reported*.

In total, 617 isolates were tested in such studies. Overall levels of phenotypic resistance were: ampicillin (median 2.3%; IQR 0.6–13.5%), gentamicin (4.3%; IQR 1.8–11.1%), erythromycin (18.0%; IQR 2.7–64.1%), florfenicol (0.6%; IQR 0–1.6%), tetracycline (13.8%; IQR 7.6–40.0%), co-trimoxazole (10.8%; IQR 0–20.0%), and enrofloxacin (4.7%; IQR 1.0–22.0%). A study on 120 isolates from poultry in India showed 100% resistance against sulfadiazine, a drug most often used in the field to treat fowl cholera in that country. Only resistance against chloramphenicol, ciprofloxacin, norfloxacin, enrofloxacin, gentamicin, and lincomycin, was observed in <10% isolates, remaining the only effective therapeutic alternatives (55). However, the authors did not provide the interpretation criteria, other than “provided by the disk manufacturer.” In another study of 56 poultry isolates from Brazil, levels of resistance were highest for sulfonamides (sulfaquinoxaline) (~77%); in contrast levels of resistance against β-lactams (amoxicillin, ceftiofur), aminoglycosides (gentamicin), and macrolides (erythromycin) were <6%. In a study from the United States of 80 isolates, resistance was less than 7% against all antimicrobials. In comparison with *E. coli* and *Salmonella* isolates, *P. multocida* isolates from poultry were found to be much more susceptible to the antimicrobials tested (33).

Studies on isolates from pigs, cattle, and poultry in Europe have shown that resistance in *P. multocida* is generally mediated by small (4–7 kb size) plasmids (59, 60). A larger plasmid (pVM111) has also been shown to contain multiple genes conferring resistance against tetracyclines, sulfonamides, and streptomycin resistance (*tetR-tet*(H), *sul2*, and *strA*), supporting the hypothesis that the spread of resistance is due to horizontal transfer of plasmids rather than clonal dissemination. It is not known whether this explains the relatively lower prevalence of AMR in *P. multocida* compared with other Gram-negative bacteria (61).

### *Avibacterium* *paragallinarum*

*Avibacterium paragallinarum* (previously *H. paragallinarum*) is a capsulated, rod-shaped, Gram-negative facultative anaerobe bacterium of the Pasteurellaceae family. It is the etiological agent of infectious coryza, an acute disease of the upper respiratory tract of chickens worldwide (62).

A total of seven publications investigated phenotypic resistance in 143 *A. paragallinarum* isolates from diseased flocks in Asia (India, Thailand, Indonesia, Taiwan) (63–67), Africa (Uganda) (68), and the Americas (Mexico, Ecuador, Peru, Panama) (5). All studies used the disk diffusion test, except one where broth microdilution (64), and one where agar dilution (68) tests were used (Table 4). Initial MIC interpretative criteria (breakpoints) of resistance for *A. paragallinarum* were provided by Fales et al. (1986) and cited by Blackall (8) (Table 4). Overall levels of phenotypic resistance for the main antimicrobials tested were: ampicillin (median 38.9%; IQR 5.9–60%), neomycin (77.4%; IQR 56.2–100%), streptomycin (72.7%; IQR 62.1–88.9%), erythromycin (77.8%; IQR 69.8–86.3%), co-trimoxazole (44.1%; IQR 19.6–67.0%).

**Table 4:** Summary of results of 7 phenotypic studies on antimicrobial resistance of *A. paragallinarum* from poultry.

Study	Reference (country)	Year of study	No. isolates	Testing method	Interpretation	Phenotypic resistance
1	(63) (Indonesia)	1991–1999	14	Disk diffusion^a^	NCCLS (1984)	AMP (7.1% R), NEO (71.4% R), S (78.6% R), ERY (78.6% R), OTC (57.1% R), DOX (35.7% R), SXT (21.4% R)
2	(64) (Taiwan)	1990–2003	18	Broth micro dilution	(8)	AMP (38.9% R), NEO (83.3% R), S (88.9% R), ERY (77.8% R), SXT (83.3% R)
3	(68) (Uganda)	Not given	5	Agar dilution	(8)	AMP (60% R), NEO (0%), S (60% R), C (0%), TE (80% R), SMX (60% R)
4^b^	(65) (Thailand)	1990–2009	18	Disk diffusion	CLSI M31-A3 (2008)	PEN (27.8% R; 27.8% I), CLX (100%), AMP (33.3% R; 5.5% I), AMX (27.8%), AMC (0%), CEF (27.8%), NEO (100%); CN (5.5% R; 11.1% I), SPC (11.1%), ERY (77.8% R; 16.7% I), TYL (0% R; 5.5% I), LCM (100%), OTC (55.6% R; 5.5% I), DOX (38.9% R; 11.1%), SXT (66.7%), ENR (27.8% R; 11.1% I)
5^b^	(66) (Thailand)	1990–2009	18	Broth micro dilution^a^	CLSI M100-S21 (2011) and (8)	AMP (5.6% R), AMX (0%), CEF (5.6% R), ERY (66.7% R), CN (55.6% R), S (66.7% R), SPC (50% R), OTC (72.2% R), DOX (66.7% R), SXT (66.7% R), CIP (66.7% R), ENR (50% R)
6	(5) (Mexico, Ecuador, Peru, Panama)	Not given	66	Disk diffusion^a^	(65)	PEN (26.0% R), AMC (4.7% R), AMP (5.9% R), S (62.1% R), CN (46.8% R), NEO (56.2% R), KA (24.5% R), ERY (73.0% R), LCM (81.5% R), TE (37.8% R), SXT (19.6% R), COL (22.8% R), FOM (1.6% R)
7	(67) (India)	Not given	4	Disk diffusion	Not given	AMP (100% R), AMC (0%), NEO (100% I), S (100% I), C (0%), TE (100% R), OTC (100% R), DOX (100% R), SXT (0%), FUR (100% I), ENR (0%), CIP (0%), PEF (0%)

*In studies where intermediate susceptibility is given, results are presented as: R, fully resistant; I, intermediate resistant*.

*CLSI, Clinical Laboratory Standard Institute; NCCLS, National Committee for Clinical Laboratory Standards; AMP, ampicillin; AMC, amoxicillin/clavulanic acid; AMX, amoxicillin; C, chloramphenicol; CEF, ceftiofur; CIP, ciprofloxacin; CLX, cephalexin; CN, gentamicin; COL, colistin; DOX, doxycycline; ENR, enrofloxacin; ERY, erythromycin; FOM, fosfomycin; FUR, furazolidone; KA, kanamycin; LCM, lincomycin; NEO, neomycin; OTC, oxytetracycline; PEN, penicillin; S, streptomycin; SMX, sulfamethoxazole; SPC, spectinomycin; SXT, co-trimoxazole; TE, tetracycline; TYL, tylosin*.

*^a^MIC distributions reported; disk concentrations reported*.

*^b^Same strain collection (year of study provided by the author as a personal communication)*.

A comparison of results between the disk diffusion method (65) and the broth microdilution (66) on the same panel (18 isolates) from Thailand revealed important discrepancies in the interpretation of results, notably for ampicillin (33.3% disk diffusion vs. 5.6% broth microdilution), amoxicillin (27.8 vs. 0%), ceftiofur (27.8 vs. 5.6%), enrofloxacin (27.8 vs. 50.0%), and spectinomycin (11.1 vs. 50.0%).

A study on isolates from Latin American countries (5) showed the lowest level of resistance against co-trimoxazole (potentiated sulfonamide). However, the authors remind that sulfonamides should be administered with caution in poultry given their low safety margin and the presence of residues in meat and eggs for a relatively longer period (13).

A study of four *A. paragallinarum* isolates in Tanzania detected genes associated with streptomycin (*strA*), ampicillin (*bla*_TEM_), tetracycline (*tetC* and *tetA*), and sulfamethoxazole (sul2) resistance (68). In a study of 18 isolates from Taiwan about 72% isolates contained plasmids pYMH5 and pA14 (64). Sequencing data indicated that pYMH5 encodes functional streptomycin-, sulfonamide-, kanamycin-, and neomycin-resistance genes (*sul2, strA, mbeCy, aphA1*).

### *Gallibacterium* *anatis*

*Gallibacterium anatis* biovar haemolytica is a Gram-negative bacterium of the Pasteurellaceae family. The organism is known to colonize the upper respiratory tract and lower reproductive tract of chickens, but also been experimentally shown to induce clinical infection (69). *G. anatis* has previously been misclassified as *M. haemolytica, P. hemolytica, P. anatis*, and *Actinobacillus salpingitidis*, but was recently classified as a new genus (*Gallibacterium*) (70). Surveillance data from the state of Mississippi (US) confirmed a progressive increase in confirmed cases of *G. anatis* from 2006 to 2011. By 2011, the annual number of confirmed cases of disease (28) was comparable with those of fowl cholera (32) (52). A total of three studies have investigated phenotypic resistance in *G. anatis* (34, 52, 71). However, in one of them, these isolates were identified as *M. haemolytica* (34). However, in the absence of specific breakpoints published, one study only indicated the mean inhibition zone of isolates, indicating that isolates showed maximum sensitivity to norfloxacin (32 mm) and minimum (16 mm) to erythromycin (71). Generally, levels of resistance were higher than those observed for *P. multocida* and *A. paragallinarum* (Table 5).

**Table 5:** Summary of results of two phenotypic studies on antimicrobial resistance of *G. anatis* and *M. haemolytica* from poultry.

Study	Reference (country)	Year of study	No. isolates	Testing method	Interpretation	Phenotypic resistance
1	(34) (United States)^a^	1998–1992	92	Disk diffusion^b^	NCCLS M31-A2 (2002)	PEN (92.4%), AMP (5.4%), CEF (0%), CN (1.1%), AK (0%), SPC (73.9%), ERY (100%), CLD (100%), TE (93.5%), SDX (85.4%), SXT (0.9%), ENR (1.3%)
2	(52) (United States)	2006–2011	84	Broth microdilution	CLSI M31-A2 (2002)	PEN (70.0% R), AMX (36.0% R; 21.0% I), CEF (3.0% R; 7.0% I), S (21.0% R; 4.0% I), NEO (14.0% R; 22.0% I), CN (4.0% R; 3.0% I), NOV (100% R), ERY (43.0% R; 57.0% I), TYL (100% R), CLD (97.0% R), SPC (0% R; 89.0% I), FFN (3.0% R; 11.0% I), TE (90.0% R; 3.0% I), OTC (83.0% R; 3.0% I), STZ (8.0% R; 10.0% I), SDX (43.0% R; 14.0% I), SXT (3.0% R; 14.0% I), ENR (4.0% R; 3.0% I)

*In studies where intermediate susceptibility is given, results are presented as: R, fully resistant; I, intermediate resistant*.

*CLSI, Clinical Laboratory Standard Institute; NCCLS, National Committee for Clinical Laboratory Standards; AK, amikacin; AMP, ampicillin; AMX, amoxicillin; CEF, ceftiofur; CLD, clindamycin; CN, gentamicin; ENR, enrofloxacin; ERY, erythromycin; FFN, florfenicol; NEO, neomycin; NOV, novobiocin; OTC, oxytetracycline; PEN, penicillin; S, streptomycin; SDX, sulfadimethoxine; SPC, spectinomycin; STZ, sulfathiazole; SXT, co-trimoxazole; TE, tetracycline; TYL, tylosin*.

*^a^Strains identified as M. haemolytica*.

*^b^MIC distributions reported;.disk concentrations reported*.

### *Ornithobacterium rhinotracheale* (ORT)

*Ornithobacterium rhinotracheale* is a Gram-negative, rod-shaped bacterium that causes respiratory disease in turkeys, chickens, and other avian species. It was first identified in turkeys in the 1990s (72). Establishing the antibiotic sensitivity of this pathogen is difficult because of its complex growth requirements. ORT is known to be often resistant to many antimicrobials, and therefore only isolates from wild birds are likely to display the highest degree of susceptibility; therefore, antimicrobial susceptibility results in these isolates have often been used to compare with those from poultry isolates (73).

Four studies investigated phenotypic resistance on ORT isolates from the Netherlands (74), Belgium (75), Hungary (76), and the Unites States (77) in a total of 600 isolates. The overall prevalence of resistance of such studies were: ampicillin (median 40.0%; IQR 11.3–100%), ceftiofur (63.0%; IQR 50.0–100%), tetracycline (21.0%; IQR 20.0–61.0%), co-trimoxazole (89.0%; IQR 25.0–97.0%), and enrofloxacin (70.8%; IQR 33.4–93.6%) (Table 6).

**Table 6:** Summary of results of 7 phenotypic studies on antimicrobial resistance of *S*. *Ornitobacterium rhinotracheale* (ORT) and *B. avium* from poultry.

Study	Reference (country)	Year of study	No. isolates (host type)	Testing method	Interpretation	Phenotypic resistance
**(A) Summary of prevalence of phenotypic resistance of ORT from poultry**						
1	(76) (Hungary)	2009–2013	36 (turkeys, chickens, pigeons)	Disk diffusion^a^, broth micro dilution, for AMX, DOX, and ERY^a^	CLSI M31-S1 (2004), CLSI M100-S21 (2011)	PEN (30.0% R; 46.7% I), AMP (40.0% R; 23.3% I), AMX (40.0% R; 23.3% I), CEF (63.3% R; 0% I), CN (100% R; 0% I), SPC (0% R; 0% I), C (0% R; 0% I), OTC (20.0% R; 20.0% I), DOX (30.0% R; 16.5% I), ERY (66.7% R; 3.3% I), LCM (70.0% R; 0% I), TIM (13.3% R; 0% I), SUL (60.0% R; 30.0% I), SXT (25.0% R; 33.3% I), NA (100% R), CIP (0% R; 70.0% I), ENR (16.7% R; 63.3% I), COL (76.7% R; 13.3% I)

2	(77) (United States)	1996–2002	124 (turkeys)	Disk diffusion	NCCLS M31-A2 (2002)	PEN (33.9%), AMP (11.3%), CEF (50.0%), CN (86.3%), ERY (0.8%), SPC (18.2%), TE (21.0%), SDM (99.2%), SCP (40.0%), SXT (96.8%), ENR (50.0%), CLD (0%)

3	(75) (Belgium)	1995–1998	45 (broilers)	Broth dilution^a^	Resistant strains had MICs over three two-fold dilution steps compared with reference strains	AMP (100%), CEF (100%), TYL (97.8%), TIM (95.5%), LCM (100%), DOX (80.0%), TIA (0%), SPI (95.5%), ENR (95.6%), FLM (93.3%)

4	(74) (Netherlands)	1996–1999	395 (broilers)	Agar dilution; Agar gel diffusion test; E-test^b^	Provided by the manufacturer	AMX (63.2%), TE (60.9%), SXT (89.3%), ENR (91.6%)

**(B) Summary of prevalence of phenotypic resistance of ***B. avium*** from poultry**						
1	(34) (United States)	1998–2002	4 (turkeys)	Disk diffusion	CLSI M31-A2 (2002)	AMP (0%), CN (0%), NEO (0%), TE (0%), SXT (0%), ERY (100% R)

2	(76) (Hungary, Germany)	1985–2012	13 (turkeys), 2 (chickens), 1 (duck), 1 (goose), 1 (partridge), 1 (unknown)	Disk diffusion^a^, broth microdilution^a^	CLSI M31-A2 (2002)	PEN (52.6% R; 47.4% I), AMP (0% R; 47.3% I), AMX (0% R; 15.8% I), CEF (100%), CN (0% R; 0% I), SPC (0% R; 0% I), ERY (57.9% R; 32.1% I), LCM (100% R), TIM (5.2% R; 0% I), C (0% R; 73.7% I), DOX (0% R; 0% I), OTC (0% R; 10.5% I), SUL (0% R; 0% I), SXT (15.8% R; 0% I), CIP (0% R; 31.6% I), NA (26.3% R; 47.4% I), ENR (15.8% R; 84.2% I), COL (0% R; 0% I)

3	(85) (United States)	Before 2011	12 (turkeys)	Broth microdilution	Levels of resistance defined in relation to the maximum dose for each antimicrobial	CTX (16.7% R; 0% I), CRO (8.3% R; 41.7% I), IMP (8.3% R; 0% I), TIC (8.3% R; 16.7% I), CAB (8.3% R; 0% I), AMP/SBM (0% R; 58.3% I), TIC/CA (0% R; 8.3% I), ATM (83.3% R; 16.7% I), TOB (8.3% R; 0% I), C (0% R; 25.5% I), TE (16.7% R; 0% I), SXT (25.0% R; 0% I), SSZ (41.7% R; 0% I), CIP (0% R; 33.0% I), LOM (8.3% R; 25.0% I), LEV (0% R; 8.3% I)

*CLSI, Clinical Laboratory Standard Institute; NCCLS, National Committee for Clinical Laboratory Standards; AMP, ampicillin; AMP/SBM, ampicillin–sulbactam; AMX, amoxicillin; ATM, aztreonam; C, chloramphenicol; CAB, carbenicillin; CEF, ceftiofur; CIP, ciprofloxacin; CN, gentamicin; CLD, clindamycin; COL, colistin; CTX, cefotaxime; CRO, ceftriaxone; DOX, doxycycline; IMP, imipenem; ENR, enrofloxacin; ERY, erythromycin; FLM, flumequine; LCM, lincomycin; LEV, levofloxacin; LOM, lomefloxacin; NA, nalidixic acid; NEO, neomycin; OTC, oxytetracycline; PEN, penicillin; SBM, sulbactam; SCP, sulfachloropyridazine; SPC, spectinomycin; SUL, sulfonamide (unspecified); SPI, spiramycin; SXT, co-trimoxazole; SSZ, sulfisoxazole; SUL, sulfonamides (unspecified type); TIC, ticarcillin; TOB, tobramycin; TE, tetracycline; TIA, tiamulin; TIM, tilmicosin; TYL, tylosin*.

*^a^Disk concentrations given; MIC distribution reported; disk concentrations reported*.

*^b^MICs provided for multi-resistant strains*.

A study determined MICs for 10 antimicrobials of 10 Mexican ORT isolates alongside 10 previously characterized strains. MIC values greater than 128 mg/mL were recorded for gentamicin, fosfomycin, trimethoprim, sulfamethazine, sulfamerazine, sulfaquinoxaline, and sulfachloropyridazine were identified among isolates. Field reports from that country confirmed that the use of gentamicin or fosfomycin had no effect when used in therapy in infected flocks, and based on these results, the authors recommended that amoxicillin, enrofloxacin, or oxytetracycline as drugs of choice (78). A study from China reported that small-colony variants, had overall higher MICs levels compared with their wild-type counterparts. Differences were also found with regards to other phenotypic characteristics, but not in their genotype (79).

A study investigated the mechanisms of enrofloxacin resistance after experimental inoculation and treatment of turkey flocks, and found that mutations in *gyrA* commonly developed after a single treatment, and it was associated with an increase in MIC (increase in MIC from 0.03 to 0.25 mg/mL), among field isolates (80).

### *Bordetella* *avium*

*Bordetella avium* is a Gram-negative, strictly aerobic bacterium, of the family Alcaligenaceae. It is the etiological agent of turkey coryza, a respiratory disease of economic importance to the turkey industry (81). In addition, the organism can however also colonize a range of wild and domestic birds (82, 83). In addition, *B. avium* organism is considered to be zoonotic, since it has been isolated from human patients with respiratory disease (84).

A total of three studies investigated phenotypic resistance in a total of 50 *B. avium* isolates, 1 by disk diffusion (from the United States) (34), 1 by broth microdilution (United States) (85), and 1 using both (Europe) (76) (Table 6). In all three studies, turkey isolates were investigated. However, one study also included chicken and pigeon isolates (76). Interpretation of results in both studies was based on criteria “for fastidious Gram-negative bacteria” (34, 76). However, in one study, the prevalence of resistance was determined in relation to the observed MICs for the antimicrobials tested (85).

A study on farmed cockatiel chicks affected with lockjaw syndrome (characterized by anorexia, sneezing, coughing, nasal discharge, and swollen infraorbital sinuses) investigated 10 isolates by disk diffusion. Isolates were sensitive to ampicillin, amoxicillin, penicillin, ceftiofur, enrofloxacin, norfloxacin, ciprofloxacin, erythromycin, florfenicol, and co-trimoxazole, whereas resistance to lincomycin and sulfadimethoxine were common to all of the isolates, and four strains showed resistance to tetracycline (86).

An experiment investigated transfer of a 12–13 kb plasmid (pRAM) coding for tetracycline and sulfonamide resistance to a receptor strain. Partial DNA sequence analysis of pRAM revealed two genes for conjugation, similar to P-type conjugative transfer ATPase, TrbB, and TrbC of *Enterobacter aerogenes* (85). There is lack of data on additional mechanisms of resistance of this poultry pathogen.

### *Clostridium* *perfringens*

*Clostridium perfringens* is a Gram-positive, rod-shaped, anaerobic, spore-forming bacterium commonly found in the intestinal tract of poultry, animals, and the environment. Under certain conditions, the bacterium can multiply, causing necrotic enteritis, and cholangiohepatitis, two diseases that are responsible for heavy losses in the broiler and turkey industry worldwide (87).

A total of seven publications have investigated phenotypic resistance in 564 *C. perfringens* isolates from Belgium (88), Scandinavia (89), Egypt (90), Korea (91), Brazil (92), and Canada (93). All studies investigated chicken isolates, except one that also included isolates from turkey species (92).

Agar dilution and broth microdilution methods were used in three and two publications, respectively. In all studies, the MIC distribution of tested strains was provided. In additional to conventional antibacterial antimicrobials, a number of studies have investigated resistance against antimicrobials commonly used as growth promoters (bacitracin, avilamycin, virginiamycin) in addition to coccidiostats (i.e., salinomycin, monensin) that are also known to have activity against *Clostridium* spp. in the gut (94).

The calculated MIC50 levels for: erythromycin 2 µg/mL (IQR 2.0–5.0), tetracycline (8 µg/mL; IQR 4.5–8), bacitracin (8 µg/mL; IQR 1–128), avilamycin (0.25 µg/mL; IQR 0.25–2.0), naransin (0.25 8 µg/mL; 0.06–0.25), salinomycin 0.5 µg/mL (0.12–0.5), and monensin (0.63 µg/mL; 0.25–1.0). Susceptibility cut-offs were determined based on the observed distribution of MICs. In addition, two studies used disk diffusion methods. However, in one publication, interpretation guidelines were not provided (Table 7). Overall levels of resistance were: tetracycline (median 66.6%; IQR 41.8–70.7%), lincomycin (62.4%; IQR 33.6–81.6%), erythromycin (17.5%; IQR 0.6–100%), bacitracin (7.5%; IQR 3.0–56.0%), ampicillin (median 0%; IQR 0–3.5%), and florfenicol (0%; IQR 0–1.0%).

**Table 7:** Summary of results of 8 phenotypic studies on antimicrobial resistance of *C. perfringens* from poultry.

Study	Reference (country)	Year of study	No. isolates (host type)	Testing method	Interpretation	Phenotypic resistance
1	(88) (Belgium)	2002	44 (healthy broilers)	Agar dilution^a^	Based on observation of MIC distributions	AMX (0% R), TYL (0% R), LCM (63.3% R), CTC (65.9% R), OTC (65.9% R), FLA (0% R), AVI (0% R), NAR (0% R), MAD (0% R), SAL (0% R), LAS (0% R), MON (0% R)
2	(93) (Brazil)	Not specified	55 (healthy broilers)	Agar dilution^a^	Based on observation of MIC distributions	PEN (0% R), LCM (3.6% R; 7.3% I), TE (41.8% R; 18.2% I), BAC (49.1% R; 43.6% I), NAR (0% R), MON (0%), AVI (0% R)
3	(92) (Canada)	2005	100 (diseased chickens)	Broth microdilution^a^	Based on observation of MIC distributions	PEN (0%), CLD (0%), BAC (64%), VIR (25%), ERY (2%), FFN (0%), TE (62%), MET (1%)
			50 (diseased turkeys)	Broth microdilution^a^	Based on observation of MIC distributions	PEN (0% R), CLD (2.0% R), BAC (60.0% R), VIR (8.0% R), ERY (0% R), FFN (0% R), TE (88.0% R), MET (0% R)
4	(91) (Korea)	2010–2012	17 (chickens, turkeys, wild birds) (suspect of necrotic enteritis)	Disk diffusion^a^	Not provided	PEN (0% R), AMP (0% R), AMC (0% R), CFN (0% R), CEF (0% R), FOX (0% R), S (100% R), NEO (100% R), CN (100% R), CLD (55.0% R), ERY (5.0% R), C (0% R), FFN (0% R), TE (45.0% R), SXT (5.0% R), SSZ (5.0% R), BAC (12.0% R), APR (100% R), COL (100% R)
6	(89) (Sweden, Denmark, Norway)	2000–2001	102 (broilers layers and turkeys) (unknown status)	Broth microdilution^a^	Based on observed MIC distributions	AMP (0% R), NAR (0% R), ERY (100% I), OTC (38.3% R), VIR (3% R), BAC (6.0% R), AVI (100% I), VAN (0% R)
7	(90) (Egypt)	2009–2010	125 (broilers) (unknown status)	Disk diffusion^a^	BSAC guidelines (2011)^b^	AMP (7.0% R), AMX (7.0% R), S (100% R), CN (100% R), NEO (93.0% R), SPC (50.0% R), ERY (100% R), LCM (100% R), PEF (94.0% R), SXT (98.0% R), OXA (100% R), SPI (100% R), FOM (2.0% R), FFN (2.0% R), CED (3.0% R), COL (94.0% R)
8	(95) Belgium	2007	71 (healthy broilers)	Agar dilution^a^	Based on observed MIC distributions	AMP (0% R), ERY (0% R), LCM (61.5% R), TYL (0% R), TE (66.6% R), FFN (0% R), ENR (0% R), BAC (0% R)

*AMC, amoxicillin/clavulanic acid; AMP, ampicillin; AMX, amoxicillin; APR, apramycin; AVI, avilamycin; BAC, bacitracin; C, chloramphenicol; CED, cefradine; CEF, ceftiofur; CFN, cephalothin; CLD, clindamycin; CN, gentamicin; COL, colistin; CTC, chlortetracycline; ENR, enrofloxacin; ERY, erythromycin; FFN, florfenicol; FLA, flavomycin; FOM, fosfomycin; FOX, cefoxitin; MET, metronidazole; LAS, lasalocid; LCM, lincomycin; MAD, maduramycin; MON, monensin; NAR, naransin; NEO, neomycin; OTC, oxytetracycline; OXA, oxalinic acid; PEF, pefloxacin; PEN, penicillin; S, streptomycin; SAL, salinomycin; SPI, spiramycin; SPC, spectinomycin; SSZ, sulfisoxazole; SXT, sulfamethoxazole/trimethoprim; TE, tetracycline; TYL, tylosin; VAN, vancomycin; VIR, virginiamycin*.

*^a^MIC distribution reported; disk concentrations given*.

*^b^Intermediate strains were considered susceptible*.

A study from Belgium on isolates collected during 2007 found high (60–70%) levels of resistance against lincomycin and tetracycline, but susceptibility to six other antimicrobials tested. However, the authors found no evidence of increases in the prevalence resistance against these antimicrobials compared with the earlier period 1980–2004 (95).

A study from Taiwan reported MIC50 values of erythromycin and lincomycin for *C. perfringens* isolated from intestinal samples with severe lesions were significantly higher compared with those with mild lesions (96). However, a study from Korea compared resistance patterns between isolates from healthy and sick flocks, and found no difference (91). Studies on *C. perfringens* isolates from Canadian chickens and turkeys had overall higher levels of resistance against bacitracin and virginiamycin compared with bovine and porcine isolates (92), but not for other antimicrobials tested.

Studies in Belgium and Scandinavia have identified *tetP(B), tet(M), tetA(P)*, and *tetB(P) genes* among tetracycline resistant isolates (88, 89). Genes *lnu(A)* and *lnu(B)* genes associated with low-level resistance against lincomycin have identified in strains from Belgium (88).

### *Mycoplasma* spp.

*Mycoplasma* spp. are Mollicutes bacteria that lack a cell wall around their membrane. *M. gallisepticum* (MG) infection is particularly important as a cause of respiratory disease and decreased meat and egg production in chickens and turkeys worldwide. Other species such as *M. synoviae* (MS) *M. meleagridis*, and *M. iowae* can also cause disease in poultry (97).

Since *Mycoplasma* spp. are fastidious organisms, routine methods based on isolation and phenotypic testing of resistance are not practicable. *Mycoplasma* spp. are unaffected by many common antibiotics that target cell wall synthesis. Antimicrobials commonly used to treat *Mycoplasma* spp. infections include tetracyclines, macrolides (tylosin, tilmicosin), and more recently, fluoroquinolones (enrofloxacin, difloxacin), and pleuromutilins (tiamulin).

A total of five studies have determined MICs among a total of 145 MG and 43 MS field strains, all using broth microdilution. Studies were carried out in Israel (98, 99), Jordan (100), Iran (101), and Thailand (102). One study compared MIC results using broth microdilution, agar dilution, and E-test methods (98). MIC data were not converted into prevalence of resistance due to the lack of published standards. For those antimicrobials included in at least three studies, the median MIC50 values (and IQRs) were, in decreasing order: erythromycin (8.8 µg/mL; IQR 0.05–128), chlortetracycline (2.73 µg/mL; IQR 1.0–4.0), enrofloxacin (1.48 µg/mL; IQR 0.26–11.31), tylosin (0.125 µg/mL; IQR 0.015–0.33), and doxycycline (0.062 µg/mL; IQR 0.015–0.2).

*In vitro* studies involving passages in sub-inhibitory concentrations of antimicrobials have shown resistance to macrolides can be quickly acquired among poultry *Mycoplasma* spp., whereas resistance to enrofloxacin develops more gradually. No resistance to tiamulin or oxytetracycline could be evidenced in MG or MS after 10 passages, whereas *M. iowae* resistant mutants were obtained. *Mycoplasma* spp. mutants that became resistant to tylosin were also resistant to erythromycin, whereas mutants made resistant to erythromycin were not always resistant to tylosin (103).

A study on MG and MS isolated from chickens and turkeys in Israel collected during 2005–2006 indicated a reduction in susceptibility against fluoroquinolones (enrofloxacin and difloxacin) compared with archived strains (1997–2003) (98). Similarly, a study from Jordan compared MICs in isolates collected from 2004 to 2005 vs. strains collected during 2007–2008 confirmed a significant increase in MIC against 8 (erythromycin, tilmicosin, tylosin, ciprofloxacin, enrofloxacin, chlortetracycline, doxycycline) of 13 antimicrobials tested (100). A study on 20 MG isolates from Thailand where MG isolates were further characterized into groups (A, B, C, D, U) by random amplification of polymorphic DNA reported the lowest MICs for doxycycline, tiamulin, and tylosin among all tested drugs. Some MG isolates low-level resistant to josamycin and were resistant to enrofloxacin and erythromycin (102).

Tiamulin (pleuromutilin) has been found in general to be a useful drug in the treatment and control of *Mycoplasma* spp. infection. However, administering tiamulin to flocks medicated with ionophore antimicrobials is not recommended, since it may lead to toxicity (104).

Fluoroquinolone resistance in *Mycoplasma* spp. is of great concern, since enrofloxacin is often the drug of choice to treat infections in poultry. However, a study showed that treatment with enrofloxacin did not succeed in eradicating infection from flocks subjected to experimental infection (105).

Resistant mutants of MG were selected *in vitro* by passaging have been shown to be due to amino acid substitutions in the *gyrA, gyrB, parC*, and *parE* genes (106, 107). A study on 93 strains from several countries indicated that MG strains with substitutions in the quinolone resistance-determining regions (QRDRs) of both *gyrA* and *parC* are resistant to enrofloxacin, however in 10% strains with such substitutions did not show a clear correlation with the MIC. The authors concluded that this may limit the applicability of a gene-based assay to detect fluoroquinolone resistance in this avian pathogen (108).

## Other Pathogens

*Erysipelothrix rhusiopathiae* is a Gram-positive, non-spore-forming, non-acid-fast, and bacillus. The organism was first identified as a human pathogen late in the nineteenth century, causing erisipeloid, a generalized cutaneous form, as well as a septicemic form often associated with endocarditis (109). The organism may cause severe disease outbreaks in a range of species including poultry and pigs (110). There are no published guidelines on interpretation of MIC or diffusion tests for *E. rhusiopathiae* and data on AMR from poultry isolates are very limited. A study in Sweden determined the MIC on 45 isolates from poultry, pigs, emus, and red mites using the broth microdilution. Although data were not presented separately by species, most isolates had a similar resistance pattern. For most of the antimicrobial agents tested, including penicillin and oxytetracycline, the MICs were low. In contrast, the aminoglycosides gentamicin, neomycin, and streptomycin had uniformly MIC levels that were greater than or equal to the highest concentration tested (111).

*Riemerella anatipestifer* is a Gram-negative, non-motile, non-spore-forming, and rod-shaped bacterium that can infect domestic ducks, geese, turkeys, and other avian species. In ducks, it causes infectious serositis, air-saculitis, meningitis, salpingitis, or septicemia with high mortality rates (112). A total of five studies investigated MICs on 481 *R. anatipestifer* strains, three from China (113–115), India (116), and one from Taiwan (117). All studies have used agar dilution method, except for one that used the disk diffusion test (113) and one where the method was not indicated (116). Based on MIC90 values, the five most potent antibacterials from Taiwan were (in descending order): penicillin, ceftiofur, cephalothin, chloramphenicol, flumequine, and kanamycin, nalidixic acid, nitrofurantoin, amikacin, ampicillin, gentamicin, lincomycin, spectinomycin, streptomycin, tetracycline, and trimethoprim (117).

A study from China investigated MICs and mutant prevention concentrations (MPC) for four antimicrobials (ceftiofur, cefquinome, florfenicol, and tilmicosin) 98 and 7 isolates from ducks and geese, respectively. Although the highest MIC values were reported for florfenicol and tilmicosin (both 1 µg/mL), followed by ceftiofur (0.063 µg/mL) and cefquinome (0.031 µg/mL), the difference between MIC and MPC values suggested that cefquinome was the drug that presented the highest risk of selecting mutant strains (114). Another study from the same country investigated antimicrobial susceptibility among 224 duck isolates, and interpreted results by observing distribution of inhibition zones using WhoNet software. Fifty percent of the isolates were resistant against ceftazidime, aztreonam, cefazolin, cefepime, cefuroxime, oxacillin, penicillin G, rifampicin, and co-trimoxazole. The authors inoculated a multi-resistant isolate with high virulence to inoculate to experimental groups, followed by subcutaneous treatment with different antimicrobial drugs. Results suggest a good correlation in the mortality with disk sensitivity results (113).

The antimicrobial susceptibility against 23 antimicrobial agents was investigated in 103 *R. anatipestifer* isolates obtained from Chinese ducks during 2008 and 2010 using agar dilution. The MIC50 and MIC90 values of streptomycin, kanamycin, gentamicin, apramycin, amikacin, neomycin, nalidixic acid, and sulfadimidine were relatively higher than for ampicillin and florfenicol (115).

A study from China has identified the presence of genes and integrons coding for resistance against β-lactamase, aminoglycoside, resistance genes, chloramphenicol, florfenicol, tetracycline, and sulfonamide resistance genes in variable frequencies. Mutation analysis of the QRDRs of identified mutations in *gyrA* responsible for quinolone resistance (115). Molecular studies have focused on the identification of macrolide resistance (118). Another study demonstrated the role of efflux pumps in multi-resistance in *R. anatipestifer* (119).

## Summary of AMR Phenotypic Data for Main Pathogens

**Figure 1:**
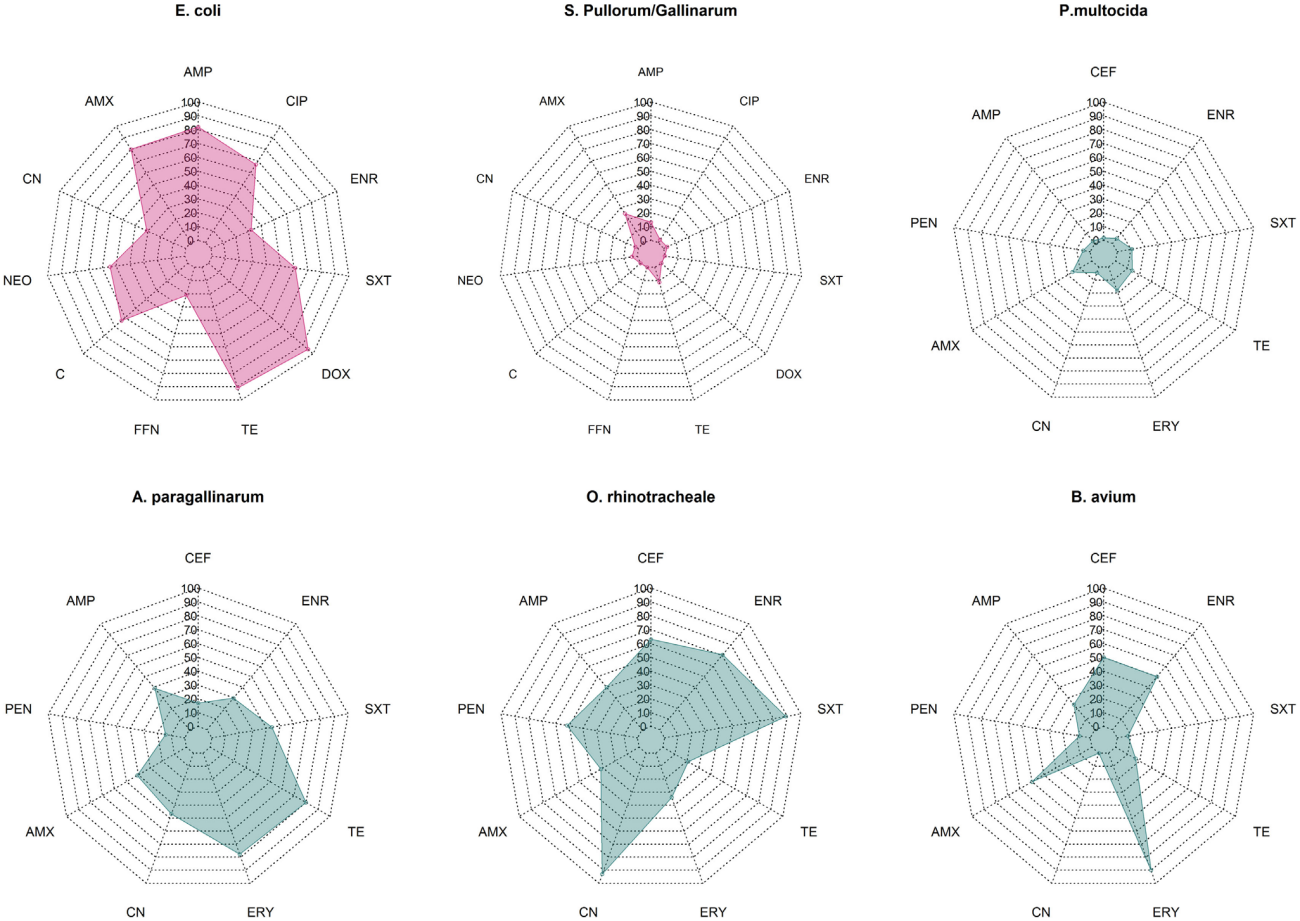
Summary data on prevalence of phenotypic resistance among in common bacterial poultry pathogens (*E. coli, S. pullorum/gallinarum, P. multocida, A. paragallinarum, O. rhinotracheale*, and *B. avium*). AMC, amoxicillin/clavulanic acid; AMP, ampicillin; C, chloramphenicol; CEF, ceftiofur; CIP, ciprofloxacin; CN, gentamicin; DOX, doxycycline; ENR, enrofloxacin, ERY, erythromycin; FFN, florfenicol; NEO, neomycin; PEN, penicillin; SXT, co-trimoxazole; TE, tetracycline.

Overall median phenotypic results across studies for six pathogens for which there are sufficient phenotypic data are presented in Figure 1. Among Enterobacteriaceae, *E. coli* displayed consistently higher levels of resistance against most antimicrobials tested compared with *S*. Pullorum/Gallinarum. Median levels of resistance against ampicillin, amoxicillin, and tetracycline and doxycycline were all >70%. Levels of resistance against ciprofloxacin, neomycin, and chloramphenicol ranged between 50 and 70%, and for gentamicin, florfenicol, and enrofloxacin ranged between 20 and 50%. In contrast, among *S*. Pullorum/Gallinarum, observed resistance levels were less than 20% for all antimicrobials, except for amoxicillin (24.8%). Among organisms with the family Pasteurellaceae, *A. paragallinarum* had the highest levels of resistance, with resistance levels greater than 70% for erythromycin and tetracycline, and resistance levels against penicillin, gentamicin, co-trimoxazole, and enrofloxacin were in the 20–50% region. In contrast, for *P. multocida*, the highest level of resistance was observed for erythromycin (18%), and levels of resistance against all other antimicrobials were <15%. Notably, ORT isolates had high levels of AMR: over 70% resistance for enrofloxacin, gentamicin, and co-trimoxazole, between 50 and 70% for ceftiofur and amoxicillin, and between 20 and 50% resistance against penicillin, ampicillin, erythromycin, and tetracycline. Among *B. avium* isolates, the levels of resistance against cephalosporin, penicillin, erythromycin, and enrofloxacin were >50%, but <25% for all other antimicrobials tested. Phenotypic resistance data from all studies has been compiled in Excel and are available in Table S1 in Supplementary Material.

## Discussion

We reviewed 70 publications published since the year 2000 containing phenotypic/genotypic data on AMR in poultry pathogens. This figure is relatively modest, compared with 196 publications returned from a search of titles including [*Salmonella* OR *Campylobacter*], AND [poultry OR chickens] AND [antimicrobial resistance OR antimicrobial susceptibility] over the same period, and 76 publications resulting from a search where [*Salmonella* OR Campylobacter] are replaced with [*Escherichia coli* OR *Enterococcus*]. A total of 13/70 (18.6%) of the reviewed publications were not indexed in MEDLINE (the bibliographic citation database of NLM’s PubMed system) (https://www.ncbi.nlm.nih.gov/pubmed), probably reflecting less stringent publication criteria for some of these journals.

There are important gaps in the knowledge on AMR in important zoonotic pathogens such as *C. psittaci* and *M. avium* detected from sick poultry. Data from isolates from human patients with chlamydiasis indicate a high prevalence of macrolide and tetracycline resistance, both of which are extensively used in poultry production (120). AMR in *M. avium* infections is also of great concern, because often drug regimens commonly used for treating tuberculosis in humans are not effective (121). However, most antimicrobials used to treat human cases of *M. avium* infection are not normally used in animal production.

Our data suggest very variable phenotypic antimicrobial susceptibility results for the same organisms across studies, which is likely to reflect differences in both AMU patterns and in testing methodologies. However, in spite of this variability, there are trends for specific organisms, suggesting that the development of AMR may also have a biological basis. Studies on isolates from healthy animals have shown that *E. coli* has a higher propensity to develop resistance compared with *Salmonella* spp. (122).

It would be expected that situations of high usage levels of antimicrobials for disease prophylaxis and growth promotion may give advantage to the transmission of organisms with higher levels of resistance (123). However, data on disease incidence of bacterial pathogens are generally missing except in a few countries, where laboratory-confirmed diagnostic surveillance data are regularly published (124).

Currently, among the international organizations, only the Clinical and Laboratory Standards Institute (CLSI) has developed protocols for susceptibility testing of certain bacteria of animal origin and determination of interpretive criteria. The CLSI document “Performance Standards For Antimicrobial Disk and Dilution Susceptibility Tests for Bacteria Isolated from Animals” (3rd Edition) (VET01S) contains interpretative data for Enterobacteriaceae, *P. aeruginosa, P. multocida*, although the standards have been validated on veterinary isolates of non-poultry origin (except for enrofloxacin in Enterobacteriaceae). However, no international-approved standards are yet available for the other pathogens listed in this review. Furthermore, for most animal pathogens, the relationship between the phenotypic data (inhibition zone, MICs) and the chances of treatment success are yet to be established.

Unfortunately, in a considerable number of studies information on the testing methodology and interpretation criteria for antimicrobial susceptibility testing was insufficient. In some few cases, information on the interpretation criteria was entirely omitted. Furthermore, the data available has not been necessarily generated using harmonized methods, thereby limiting comparability across studies. A web-based platform available to researchers and practitioners that include AMR data on poultry pathogens (including testing methodologies and results, either MIC or inhibition zones) would be desirable so that field testing data could be compared with results from other areas. Such initiatives focused on animal pathogens are already taking place in the European Union with initiatives such as VetPath and Germ-Vet (125, 126), with the capacity to be integrated into national surveillance systems of AMR, provided that appropriate statistical methods are used to ensure the representativeness of isolates included (127).

Studies have shown increases in resistance over time for *S*. Pullorum/Gallinarum, MG, and *G. anatis*. However, the absence of large collections of pathogens investigated for AMR over time is a limitation for establishing the evolution of AMR. Studies on larger collection of *E. coli* strains have conclusively demonstrated increases in resistance over time against most antimicrobials in the United States (4).

Control of bacterial diseases in poultry often relies on the use of prophylactic antimicrobial treatment at different critical points during the rearing period. Given the observed prevalence of AMR it would be expected that in cases where the pathogen is resistant, the use of certain antimicrobials would result in treatment failure. It would be desirable to identify the burden of disease for each pathogen in each country, and if the disease burden justifies it, implement prophylactic vaccination. Except for *G. anatis* and *C. perfringens*, vaccines against most bacterial diseases with AMR data presented here have been developed and are available in many countries. However, most vaccination programs are strongly biased toward the prevention of viral diseases. In recent times, more research has been emerging on the potential value of using plant extracts to control bacterial diseases in poultry (128).

There is a consensus among the scientific community that excessive AMU in food animal (including poultry) production should be restrained to limit the impact of AMR on human health (129). In addition to these concerns, AMR in poultry pathogens will inevitably result in treatment failure of poultry diseases, therefore leading to increased pathogen transmission, and production losses. The magnitude of economic losses due to untreated disease has yet to be estimated, but could theoretically be calculated by integrating disease incidence, AMR, and country-wide treatment data.

In order to allow comparability of results across studies, we suggest that in the future, at the very least, all published studies on AMR in poultry pathogens should report the MIC frequency distributions (for dilution tests), disk concentration, as well as disk diffusion zones (for diffusion tests). Ideally, studies should always attach their raw data as an appendix. These distributions will enable the determination of resistance percentages, once any new interpretive criteria are made available (130).

In most countries, worldwide farming is conducted without veterinary supervision, and a wide range of antimicrobials is normally available to farmers “over the counter.” Prudent use practices should include restricting the access for use of antimicrobials that are considered to be important for human medicine in animal production (15). Such restrictions are only currently being enforced only in a number of industrialized countries (12, 131, 132). Measures such as education on good farming practices, limiting the availability of antimicrobials, and building up a knowledge base on the AMR profile of poultry pathogens will encourage responsible AMU, contributing to reduce treatment failure of poultry diseases, therefore helping reduce associated economic losses.

## Author Contributions

JC-M conceived the idea, provided the structure, wrote the introduction, the methods section, the abstract, and contributed to the discussion. NN and NC carried out the literature review and contributed to the writing up. NN compiled and summarised all data from the original publications, and created the spider charts.

## Conflict of Interest Statement

The authors declare that the research was conducted in the absence of any commercial or financial relationships that could be construed as a potential conflict of interest.
